# IGF-Binding Proteins: Why Do They Exist and Why Are There So Many?

**DOI:** 10.3389/fendo.2018.00117

**Published:** 2018-04-09

**Authors:** John B. Allard, Cunming Duan

**Affiliations:** Department of Molecular, Cellular and Developmental Biology, University of Michigan, Ann Arbor, MI, United States

**Keywords:** insulin-like growth factor, insulin-like growth factor-binding protein, insulin-like growth factor 1 receptor, insulin-like growth factor signaling, evolution

## Abstract

Insulin-like growth factors (IGFs) are key growth-promoting peptides that act as both endocrine hormones and autocrine/paracrine growth factors. In the bloodstream and in local tissues, most IGF molecules are bound by one of the members of the IGF-binding protein (IGFBP) family, of which six distinct types exist. These proteins bind to IGF with an equal or greater affinity than the IGF1 receptor and are thus in a key position to regulate IGF signaling globally and locally. Binding to an IGFBP increases the half-life of IGF in the circulation and blocks its potential binding to the insulin receptor. In addition to these classical roles, IGFBPs have been shown to modulate IGF signaling locally under various conditions. Although members of the IGFBP family share significant sequence homology, they each have unique structural features and play distinct roles. These IGFBP genes also have different modes of regulation and distinct expression patterns. Some IGFBPs have been found to bind to their own receptors or to translocate into the interior compartments of cells where they may execute IGF-independent actions. In spite of this functional and regulatory diversity, it has been puzzling that loss-of-function studies have yielded relatively little information about the physiological functions of IGFBPs. In this review, we suggest that evolution has tended to retain an array of IGFBPs in order to facilitate fine-tuning of IGF signaling. We explore the emerging explanation that many IGFBP functions have evolved to allow the targeted adjustment of IGF signaling under stressful or irregular conditions, which would likely not be revealed in a standard laboratory setting.

## Introduction

The insulin and insulin-like growth factor (IGF) signaling pathway is highly conserved among the metazoans. Many invertebrates have large numbers of insulin-like peptides (ILPs); for instance, the *Caenorhabditis elegans* genome contains around 40 (http://wormbase.org), and the *Drosophila melanogaster* genome contains 8 (http://flybase.org). In vertebrates, the ancestral insulin-like gene has diverged into insulin, IGFs-1 and -2, and several ILPs including relaxin and relaxin-like peptide (1). Insulin primarily acts in an endocrine fashion to regulate metabolism, whereas IGFs have a variety of roles as endocrine, paracrine and autocrine factors that promote cell growth, proliferation, differentiation, survival, etc. Both IGF-1 and IGF-2 bind to the IGF-1 receptor (IGF1R), which is expressed in almost all cells, with hepatocytes being an important exception in mammals. The liver secretes IGF-1 into the circulation in response to growth hormone (GH) stimulation (2, 3). Local tissues also secrete IGF-1 in response to GH, and this paracrine/autocrine IGF-1 acts together with the endocrine IGF-1 (mostly liver derived) to mediate the global growth promoting effects of GH (4). In addition to their role in regulating fetal, neonatal, and postnatal growth, IGFs are also involved in diverse processes including wound healing (5), development of CNS and other tissues (6), regulation of protein, carbohydrate, and lipid metabolism (7), neuroprotection (8), aging (9), etc.

The diverse functions of this central hormonal pathway require that robust regulatory mechanisms be in place to avoid inappropriate regulation and/or dysfunction in different tissues and at different times. We now understand that IGF signaling is regulated by a family of specific IGF-binding proteins (IGFBPs) of which there are six distinct types in vertebrates. These IGFBPs share significant sequence homology and they are capable of binding IGFs with equal or greater affinity than the IGF1R. In fact, in both the circulation and in local tissues, most IGFs can be found bound to an IGFBP (10–13). In this review, we discuss the complex interplay of both overlapping and unique functions by which IGFBPs influence IGF signaling.

## The IGFBP Family

The IGFBP family is evolutionarily ancient and highly conserved in vertebrates (11, 14–16). The six types of IGFBPs have been designated IGFBP-1 through IGFBP-6. Mammals generally possess one gene that belongs to each of the six types, and humans follow this pattern (Table 1). Some vertebrate species occasionally lack one or more of the types, and others have more than one IGFBP gene that can be classified within one type (Table 1). It is believed that the IGFBP family evolved *via* successive rounds of whole genome duplications. Notably, many teleost fish possess two copies of each of the six types of IGFBPs (Table 1), which is attributable to the third round of whole genome duplication that they are believed to have undergone following their divergence from the other vertebrates (16–21). Salmonid fish experienced an additional round of genome wide duplication and can have four copies of each IGFBP (22–24). We discuss the evolution of the IGFBP family in more detail in a later section.

**Table 1:** The IGFBP gene repertoire in selected vertebrate species.

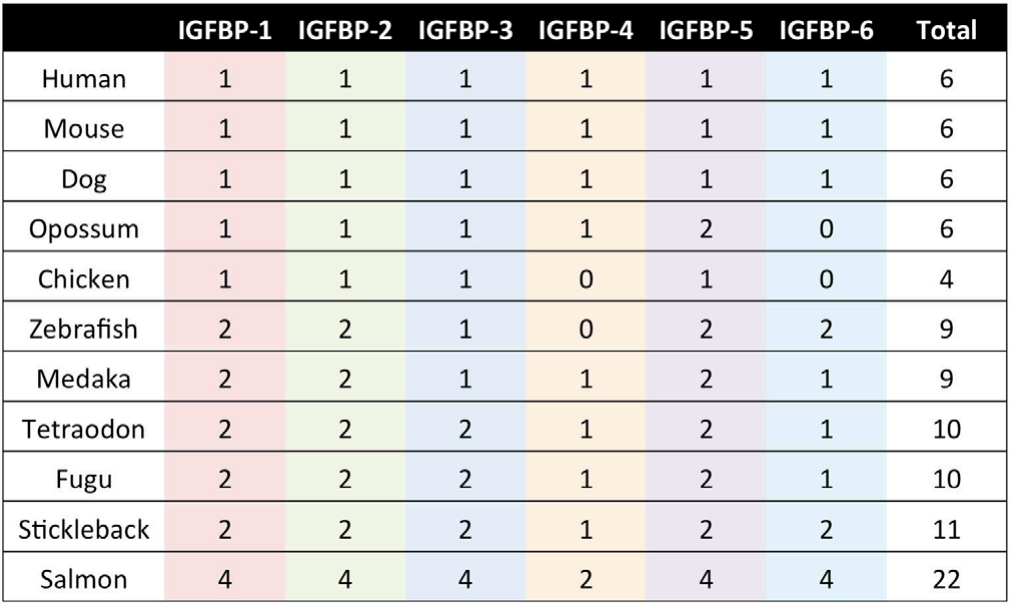

**Figure 1:**
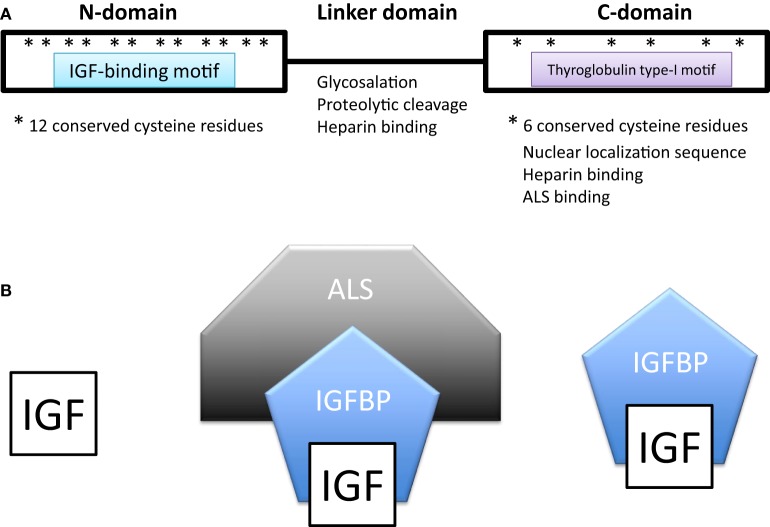
**(A)** Domain structure of insulin-like growth factor-binding proteins (IGFBPs). IGFBPs contain conserved N- and C-terminal domains and a variable linker domain between them. The N-domain contains an insulin-like growth factor (IGF)-binding motif and the C-domain contains a thyroglobulin type-I repeat. The N-domain usually contains 12 conserved cysteine residues and the C-domain contains 6. **(B)** In extracellular environments, most IGFs are bound with IGFBPs, either in a binary complex containing one IGF and one IGFBP or a ternary complex consisting of an IGF, IGFBP-3 (or less often IGFBP-5), and a glycoprotein called acid labile subunit (ALS).

All IGFBPs generally have approximately 200–300 amino acids and share a conserved structure consisting of a highly cysteine-rich N-terminal domain that is highly conserved among the IGFBP family and across species, a linker domain whose sequence is variable, and a cysteine-rich C-terminal domain that is also evolutionarily conserved (Figure 1A). The N- and C-terminal domains are globular and are structurally stabilized by multiple disulfide bonds between the conserved cysteine residues. Both of these domains participate in forming the IGF-binding site. The central linker domain is unstructured and serves to tether the N- and C-terminal domains together but also provides a location for functional motifs (10, 25).

Many of the functions of different IGFBPs are made possible by their unique collection of functional motifs (Table 2). These functional motifs include binding sites for heparin, components of the extracellular matrix and cell surface proteoglycans; proteolytic cleavage sites; sites of post-translational modifications including glycosylation, etc. In addition, IGFBP-2, -3, -5, and -6 all contain functional nuclear localization sequences by which they are imported into the cell nucleus in certain cell types as we discuss below.

**Table 2:** Structural and biochemical characteristics of the six distinct types of IGFBPs.

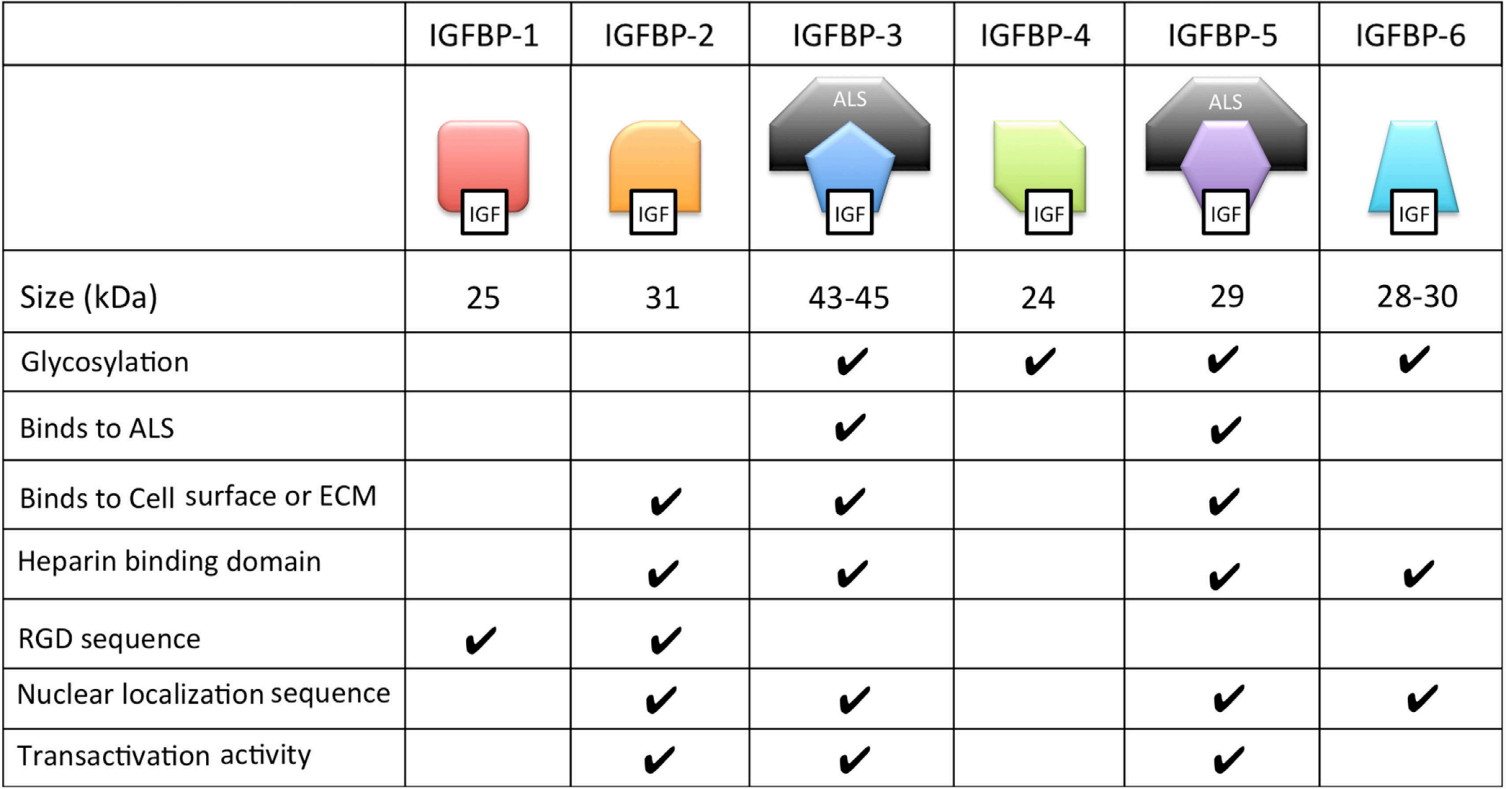

It should be noted that a number of proteins belonging to the CCN (cyr61, ctgt, and Nov) protein family have been reported to contain sequences similar to the IGFBP N-domain and were once named IGFBP 7–12. It was later recognized that these were not IGFBPs and were renamed as IGFBP-related proteins and classified as part of a broader superfamily with the IGFBPs (26). The latter nomenclature has been questioned because these CCN family proteins not only lack high-affinity IGF-binding abilities but are also structurally no more related to IGFBPs than to von Willebrand factor, thrombospondin, or growth factor cysteine knots (27). These CCN family proteins will not be discussed in this review.

## IGFBP Biological Actions

In this section, we discuss a selection of the vast literature on the many reported biological actions/activities of IGFBPs that have been reported in gain-of-function studies *in vivo* or *in vitro*. Each species has its own standard nomenclature for gene and protein names. In this article, we deal with a large number of species. To increase the readability, we will use the same symbol for each IGFBP name. Whenever required, the species name is added to avoid confusion.

### Endocrine Actions of IGFBPs

In extracellular environments, most IGFs are bound with IGFBPs, either in a binary complex or a ternary complex (Figure 1B). The vast majority of IGFs in the serum are bound to an IGFBP. IGFBP-3 is the most prevalent in adult serum with a concentration of around 100 nM/L, while all of the other IGFBPs are present at concentrations of less than 20 nM/L (25, 28). About 75–80% of serum IGFs were found in a ternary complex of about 150 kDa consisting of an IGF, IGFBP-3 (or less often IGFBP-5) and a glycoprotein called acid labile subunit (ALS). The remaining 20–25% of IGFs were complexed with one of the other IGFBPs (25, 28). Unbound IGFs have a half-life of less than 10 min (29). Binding to an IGFBP increases IGF half-life in the circulation to around 25 min, but the binary complexes are able to rapidly leave the circulation (29). Most of the circulating IGFs are present in the IGF-IGFBP3/5-ALS ternary complex (30, 31). The addition of ALS increases the molecular size of the complex and this has the effect of preventing the bound IGF from leaving the capillaries thereby confining it within the circulation (32). The ternary complex thereby greatly prolongs the half-life of bound IGFs to about 16 h or more, forming a long-lasting reservoir of IGFs in the circulation (28). Deletion of the ALS gene in mice results in a 60% reduction in circulating IGFs and a 15–20% reduction in postnatal growth (33).

Insulin-like growth factors are sufficiently structurally similar to insulin that they can cross react with the insulin receptor (IR) (34). Another important function of circulating IGFBPs is to prevent the potential interaction of IGFs with the IR, which is crucial since IGF concentrations are high enough in the serum to cause hypoglycemic effects even given their lower affinity for the IR (25, 35).

Insulin-like growth factor-binding protein-3 is produced in the liver and in other tissues and secreted into the serum, and its hepatic expression level is regulated by GH (36). This ensures that as the amount of secreted IGF-1 increases in response to GH stimulation, there will be an increased quantity of IGFBP-3 to absorb it in the circulation. IGFBP-1 is also synthesized in the liver and its expression and secretion are highly regulated by catabolic factors and hormones. For example, hepatic IGFBP-1 expression level is highly induced by starvation, hypoxia, and stress (37, 38). Its expression is reduced by insulin and increased by glucocorticoids (39, 40). These regulatory mechanisms serve to promote IGFBP-1 expression in response to starvation and catabolic conditions, including amino acid shortages and hypoxia (41, 42). The functional role of IGFBP-1 in these conditions is to reduce the rate of development and growth by binding to IGFs and inhibiting IGF activity (37, 38).

### Local Actions of IGFBPs

**Figure 2:**
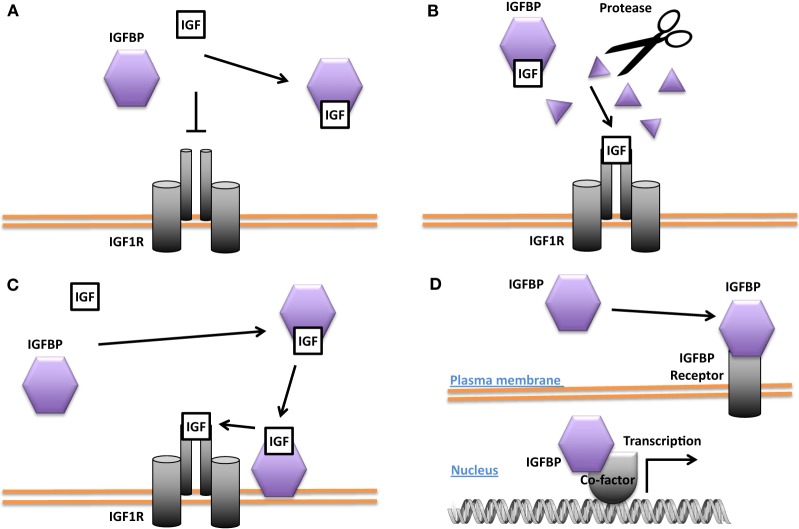
Different modes of Insulin-like growth factor-binding protein (IGFBP) actions. **(A)** Inhibition of insulin-like growth factor (IGF) signaling by sequestering IGFs away from the IGF-1 receptor (IGF1R). **(B)** Promotion of IGF signaling by proteolytic cleavage of the IGFBP and liberation of IGFs from the IGF/IGFBP complex for binding to the IGF1R. **(C)** Enhancement of IGF signaling by concentrating IGF locally and increasing IGF availability for binding to the IGF1R. **(D)** IGF-independent actions. Some IGFBPs have been shown to be capable of translocating into the nucleus in certain cells and may affect gene transcription directly or indirectly. Some IGFBPs have been found to bind to cell surface proteins that may act as IGFBP receptors.

While the bulk of circulating IGFBP-3 and IGFBP-1 are produced in the liver, IGFBP-3 and other IGFBPs are also expressed in many peripheral tissues (43, 44). The importance of local IGF-1 is supported by the finding that deletion of IGF-1 specifically in the liver resulted in an 80% reduction in circulating endocrine IGF-1 but no change in postnatal growth (45). Biochemical and cell culture studies suggest that IGFBPs generally bind IGFs with equal or higher affinity than the IGF1R and can inhibit IGF signaling by sequestration of the ligands (12, 13, 25, 46) (see Figure 2A). An example of this behavior is found in vascular smooth muscle cells (VSMCs) where IGFBP-4 acts to block IGF-1 from interacting with the IGF1R and thereby inhibits IGF-1-stimulated DNA synthesis (47). When IGFBP-4 was overexpressed in various tissues in mice, it resulted in hypoplasia of the affected tissue, suggesting that this may be a common action in different cell types (48).

Some IGFBPs have been shown to potentiate IGF signaling. Several proteases are known to cleave IGFBPs, and the resulting proteolytic fragments have greatly reduced binding affinity for IGFs. This leads to the liberation of IGFs from the IGF/IGFBP complex and increases the amount of IGFs available for IGF1R binding, thereby converting the inhibition of IGF signaling into an enhancement (Figure 2B). The proteases pregnancy-associated plasma protein A (PAPP-A) and PAPP-A2 are specific IGFBP proteases (49). IGFBP-4 is cleaved by PAPP-A when bound to IGF. This results in the release of the IGF ligand from the complex and a consequent increase in IGF ligand available for binding to the IGF-1R (50). PAPP-A knockout mice were about 40% smaller than wild-type littermates, which is consistent with the idea that PAPP-A cleaves inhibitory IGFBPs and thereby promotes IGF action (51). IGFBP-4 knockout mice were paradoxically slightly smaller than wild-type littermates, but mice null for both IGFBP-4 and PAPP-A were not smaller than IGFBP-4 knockout mice, indicating that the growth-promoting effects of PAPP-A likely result from the cleavage of IGFBP-4 (52). A number of other proteases have also been found to cleave various IGFBPs (53–55).

The potentiating action of some IGFBPs can occur when the IGFBP binds to the target cell’s surface proteoglycans and/or extracellular matrix components, resulting in a concentration of local IGF that can then be released to the IGF1R (Figure 2C). It has been reported that IGFBP-5 undergoes a reduction of affinity for IGFs when it binds to certain extracellular matrix components, allowing it to deliver and then release IGF ligands at target sites (56, 57). Differentiating myoblasts provide one example of the interplay between a locally produced IGFBP and autocrine/paracrine IGF signaling. During myogenesis, IGF-2 is produced locally at high levels and this is required for myoblast differentiation (58). Prior to the onset of IGF-2 secretion, there is an increase in the expression and secretion of IGFBP-5. The secreted IGFBP-5 potentiates IGF-2 signaling and increases myoblast cell differentiation by binding to IGF-2 and promoting its interaction with the IGF-1R (59).

Insulin-like growth factor-binding proteins have also been reported to act locally under certain pathological conditions. A good example is the role of IGFBP-5 in the progression of atherosclerosis. IGFBP-5 is normally produced and secreted by VSMCs but its expression is upregulated in the VSMCs found within atherosclerotic plaques (60). Immunostaining for IGFBP-5 protein was dense within atherosclerotic plaques and especially around calcified areas (61). Locally secreted IGFs have been suggested to play an important role in atherogenesis by promoting the VSMC proliferation and their migration into the area of the arterial wall known as the intima (62, 63). These actions are promoted by local IGFBP-5 (57). Interestingly, a protease resistant IGFBP-5 mutant actually inhibited VSMC proliferation and migration, suggesting a mechanism by which IGFBP-5 is normally cleaved in order to present the IGF ligands to the IGF-1R on the surface of VSMCs (56). IGFBP-5 also binds to certain extracellular matrix proteins that are enriched in atherosclerotic lesions. It was reported that these interactions enhanced the mitogenic effects of IGFs on VSMCs (53, 55, 64). These studies support a model in which local IGFBP-5 is concentrated within atherosclerotic lesions (by both increased local expression and secretion and by binding to locally enriched ECM components), where it then acts to concentrate and deliver IGFs to the IGF1R on local VSMCs.

Studies suggest that IGFBP-4 may be involved in inhibiting atherosclerosis. A protease-resistant IGFBP-4 mutant was able to inhibit atherosclerotic lesion development in hypercholesterolemic pigs (65). When the PAPP-A protease that cleaves IGFBP-4 was knocked down in a mouse model of atherosclerosis (ApoE KO), there was decreased formation of atherosclerotic lesions (66, 67). When PAPP-A expression was transgenically increased locally within VSMCs in artery walls in ApoE KO mice, there was a substantial increase in atherosclerotic plaque formation that was associated with an increase in local IGF-1 availability (68). Targeting PAPP-A in ApoE KO mice with a monoclonal antibody that inhibited its proteolytic activity resulted in a 70% reduction in aortic plaque burden (69). These results suggest that the proteolysis of IGFBP-4 by PAPP-A releases IGF-1 that acts locally to promote atherosclerotic plaque formation.

### IGF-Independent Actions

Several IGFBPs have been reported to have cellular actions that are independent of their IGF binding (Figure 2D). Some IGF-independent actions are mediated by binding to cell surface proteins. For example, the integrin-binding RGD motif found in IGFBPs 1 and 2 allows them to promote cell migration and influence cell adhesion, respectively (70, 71). IGFBP-5 and -3 possess functional nuclear localization sequences and can enter the nucleus (72, 73). The nuclear localization of IGFBP-3 and -5 was found to be mediated by importin beta (72). Locally produced IGFBP-5 was found to stimulate porcine VSMC migration by an IGF-independent mechanism (74). IGFBP-5 was shown to possess transactivation activity (73). This transactivation activity was mapped to the N-domain and was also demonstrated in the N-domains of IGFBPs-2 and 3 (75). Nuclear localization and transactivation activity are also present in zebrafish IGFBP-3 and -5 (21, 76). In *cephalochordate* amphioxus, which diverged from the vertebrates approximately 520 million years ago, there is a single IGFBP-like gene. The amphioxus IGFBPs contains a functional nuclear localization signal and a transactivation domain (77). The lamprey IGFBP3, a jawless agnathan vertebrate, has been reported to possess both IGF-dependent action and the transactivation activity. The conservation of IGFBP transactivation activity across eons of evolution suggests that it likely has an important function. Along this line, several studies have found roles for nuclear IGFBPs in altering transcription in cancer cells (78, 79), but the physiological role(s) of the endogenous IGFBPs in the nucleus remain unclear.

Other IGF-independent actions have been reported that do not apparently involve nuclear localization. Paracrine IGFBP-4 was shown to promote differentiation of cardiomyocytes by inhibiting Wnt signaling in an IGF-independent manner (80). The physiological relevance of this effect was supported by the fact that knockdown of IGFBP-4 in Xenopus embryos resulted in cardiac defects attributable to impaired cardiomyogenesis (80). On the other hand, IGFBP-4 knockout mice have no cardiac phenotype (81). The lack of phenotype may be due to genetic redundancy and/or compensation by other IGFBPs. Another example is the antagonization of bone morphogenic protein signaling by IGFBP-3 in zebrafish (76). It has been reported that human IGFBP-6 has antiangiogenic activity when tested using *in vitro* assays. This action is independent from IGF binding because an IGFBP-6 mutant with 10,000-fold lower binding affinity for IGFs was as potent as the wild-type human IGFBP-6 in inhibiting angiogenesis (82). Interestingly, IGFBP-6 was found to be able to bind vascular endothelial growth factor (VEGF) and coincubation with IGFBP-6 abolished VEGF-stimulated angiogenesis. This antiangiogenic action of IGFBP-6 was demonstrated *in vivo* in a tumor model by transplanting human Rh30 rhabdomyosarcoma cells stably transfected with IGFBP-6 into BALB/c nude mice (82). Expression of zebrafish IGFBP-6b had similar effects, indicating that this antiangiogenic activity is evolutionarily conserved (82).

Some IGFBPs may have cell surface receptor-mediated IGF-independent actions (Figure 2D). Exogenous IGFBP-3 was reported to inhibit cultured cell growth by an IGF-independent mechanism (83, 84). This effect was shown to be related to the binding of IGFBP-3 to the type 5 transforming growth factor β (TFGβ) receptor (85). This receptor was then shown to be identical to the low-density lipoprotein receptor-related protein-1 (LRP-1) (86). LRP-1 is known to be responsible for the uptake and clearance of various molecules from the circulation (87). The downstream mechanisms by which the interaction of IGFBP-3 with LRP-1 may lead to growth inhibition remain unclear. IGFBP-2 has been shown to bind to a receptor called receptor protein tyrosine phosphatase β (RPTPβ), which triggers a signal transduction cascade that leads to reduced PTEN phosphatase activity and a consequent enhancement of IGF-1-induced Akt pathway activation (88). This interaction between IGFBP-2 and RPTPβ was shown to be responsible for the ability of IGFBP-2 to trigger osteoblast differentiation (89). This role of IGFBP-2 was independent of IGF-binding and a 13-residue peptide corresponding of IGFBP-2’s heparin-binding domain 1 was shown to mediate its binding to RPTPβ (88, 89).

### Loss-of-Function Studies

Given the numerous biological actions of IGFBPs found in gain-of-function studies, it was surprising that little or no phenotypic change was observed when individual IGFBP genes were deleted in mice (81, 90–93). IGFBP-1 knockout mice were indistinguishable from their wild-type littermates and no embryonic lethality was observed (91). IGFBP-2 knockout mice were phenotypically normal with the exception of minor gender specific changes in bone structure and minor changes in the weights of spleen and liver in adult males (90, 92). IGFBP-3 knockout mice were also normal (81). Deletion of the IGFBP-4 gene in mice resulted in a mild 10–15% reduction in prenatal growth, which is somewhat paradoxical given that overexpression of IGFBP-4 also reduces growth (81). IGFBP-5 knockout mice were also phenotypically normal (81). Genetic deletion of IGFBP genes using CRISPR-Cas9 or TALEN in zebrafish have also resulted in little or no alteration in phenotype. Zebrafish IGFBP-3 knockout fish are morphologically normal and their growth rate and developmental speed are comparable to their siblings. Likewise, IGFBP-5a and -5b knockout zebrafish are morphologically indistinguishable from their wild-type siblings when kept under optimized lab conditions (unpublished data).

When IGFBP-3, -4, and -5 were knockout together in mice, there was a 25% reduction in body growth, decreased fat accumulation and quadriceps muscle mass, expanded pancreatic islets, and enhanced glucose homeostasis (81). These triple mutant mice were viable (81). Considering that knockout of IGF-1 itself results in a 60% reduction in prenatal growth followed by perinatal lethality for over 95% of mutant pups (94), the phenotype of the triple IGFBP-3/4/5 knockout mice can be viewed as relatively moderate.

The lack of substantial phenotypes in these IGFBP mutant mice and the finding that these animals can survive without three out of the six IGFBPs suggests a high degree of functional redundancy and/or genetic compensatory mechanisms. Indeed, elevated levels of IGFBPs-1, -3, and -4 were found in the IGFBP-2 knockout mice, supporting the notion that the lack of IGFBP-2 may be compensated for by upregulation of other IGFBPs (90).

Genetic redundancy among paralogous genes is a widespread phenomenon and can result in the masking of phenotypes in loss-of-function studies (95, 96). One study of the *Drosophila* genome suggested that when gene duplications occur, only 4% of the resulting paralogs survive (97). One explanation for the stable retention of redundant paralogous genes is that genes with redundant functions may also acquire functions that are unique to themselves. This can result in the coselection of the redundant functions with the unique functions in a model referred to as the “piggyback” mechanism (98, 99). In this model, whenever it is the case that most mutations tend to inactivate both the redundant and non-redundant functions simultaneously, redundant functions can then be retained in both gene duplicates. Unique functions could be obtained by gain-of-function mutations, but it is more common for complementary inactivating mutations to cause ancestral functions to be partitioned between the duplicates in the process of subfunctionalization (100). Redundant functions can be maintained in both duplicates when at least one unique function is maintained in each duplicate (100).

## Why are there so many IGFBPs?

Why has evolution favored the retention of so many IGFBP genes? One potential explanation is that, given the crucial importance of the IGF pathway in determining central life history traits such as body size and longevity, it may be that even relatively minor fine-tuning of IGF signaling levels would be strongly selected for. A possible example comes from the zebrafish IGFBP genes. In zebrafish, there are two IGFBP-1 genes, being paralogs of mammalian IGFBP-1 (17). Zebrafish IGFBP-1a and -1b have similar expression patterns and regulatory responses, but IGFBP-1a has a higher affinity for IGFs than IGFBP-1b, which may allow more graded inhibition of IGF signaling during catabolic conditions than was possible with only a single IGFBP-1 gene (17). The zebrafish genome also contains two IGFBP-2 genes. In this case, the IGFBP-2a and -2b proteins have similar biological activities (18, 101). However, these two paralogous genes exhibit distinct spatiotemporal expression patterns. During embryogenesis, IGFBP-2a mRNA is found in the lens and the brain boundary vasculature; it subsequently becomes highly expressed in the liver. IGFBP-2b is detected initially in all tissues at low levels, but later becomes abundant in the liver (18). In the adult stage, liver has the highest levels of IGFBP-2a mRNA, followed by the brain. IGFBP-2b mRNA, on the other hand, is only detected in the liver (18, 101). The two zebrafish IGFBP-5 genes have diverged both in gene expression patterns and protein functions. Zebrafish IGFBP-5a and -5b are expressed in spatially restricted, mostly non-overlapping domains during early development (21). The IGF-binding site is conserved in both zebrafish IGFBP-5a and -5b, and they are both secreted and capable of IGF binding (21). While zebrafish IGFBP-5b has transactivation activity, no such activity is found with IGFBP-5a (21). Given their divergence in both expression patterns and cellular actions, zebrafish IGFBP-5a and 5b may regulate IGF-signaling within their respective domains in subtly differing ways. This may provide enhanced fine-tuning of IGF signaling as compared with a single IGFBP-5 gene.

A second possible explanation is that genetic compensation is responsible for masking what would otherwise be more significant phenotypes. It has been recognized recently that permanent genetic deletions (knockouts), often result in a less severe phenotype than transient reductions in expression (knockdowns) (102). The mechanisms responsible for this phenomenon remain unclear but a number of hypotheses have been proposed, including the idea that related or unrelated genes could be upregulated in the permanent mutants (102). When zebrafish IGFBP-3 was deleted, for example, no phenotypes were detected. However, when zebrafish IGFBP-3 was knocked down using antisense morpholinos, it resulted in defects in the development of the pharyngeal skeleton and inner ear (103).

Another possible explanation is that, in addition to their somewhat overlapping functions of transporting and protecting IGFs in the circulation, the individual IGFBPs are also involved context-dependent regulation of IGF signaling in specific cell types and under specific stressful or aberrant conditions. Flexible and versatile modes of regulation such as these would be highly advantageous for organisms in the wild and would be strongly selected for, despite being unlikely to produce observable phenotypes under optimized laboratory conditions. One example is the role of IGFBP-1 in responding to catabolic conditions by throttling back growth and developmental rate in order to conserve scarce resources (38, 42, 104). Another example is the specific role of IGFBP-1 in liver regeneration. IGFBP-1 knockout mice exhibited normal growth but were found to have impaired liver regeneration (91). Their liver cells were highly sensitive to induction of apoptosis by treatment with Fas agonist. This effect could be ameliorated by pretreatment with IGFBP-1 (105), suggesting that IGFBP-1 has a crucial but conditional role in protecting the liver when facing injury and healing. The role of IGFBP-5 in mammary gland remodeling is a further example. The IGFBP-5 knockout mice had normal body growth and normal mammary gland development under standard laboratory conditions. However, these mutant mice exhibited delayed mammary gland involution and enhanced alveolar bud formation after ovariectomy and estradiol/progesterone treatment (106). Another example is provided by zebrafish IGFBP5a, which is specifically expressed in a specific type of epithelial cell (ionocytes) on the larval yolk sac skin that are responsible for transporting Ca^2+^ ions. When wild type larvae are raised in embryo solution containing a very low calcium concentration, these ionocytes rapidly proliferate *via* a mechanism that requires the activation of IGF signaling in these cells (107). This allows increased calcium import and is necessary for survival under these conditions. This proliferation is blunted in the IGFBP5a knockout fish larvae, causing lethality. However, under optomized and calcium-rich conditions, these mutant fish are indistinguisable from their wild type siblings. This suggests that IGFBP-5a is critical for calcium ionocytes to activate a conditional proliferation program in order to maintain calcium homeostasis.

**Figure 3:**
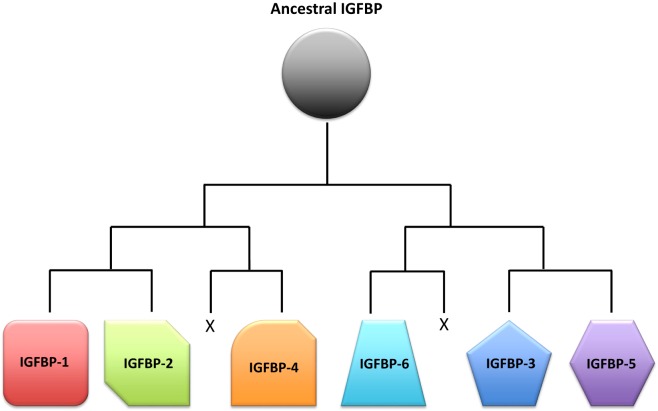
Schematic representation of a proposed scenario of the insulin-like growth factor-binding protein (IGFBP) family evolution. A single ancestral IGFBP gene was duplicated in an early chordate. This duplication was followed by two successive rounds of chromosomal duplications or tetraploidization events in early vertebrates. Of the eight IGFBPs that resulted from this process, two were subsequently lost, leaving six types of IGFBPs that are seen in modern vertebrates.

As always in biology, the question of why there are so many IGFBPs can only be fully understood in the context of evolutionary history. Based on analyses of phylogenetic relationships, the surrounding chromosomal regions in which modern IGFBPs sit, and IGFBP sequences from a large number of species, the evolution of the IGFBP family in early vertebrate ancestors has been reconstructed (16). An ancestral IGFBP sequence in an ancient early chordate was duplicated resulting in two adjacent IGFBP sequences in a chromosomal region that also bore the homeobox (HOX) genes. These two original genes were then duplicated along with the entire genome in the two successive rounds of tetraploidization that occurred in early vertebrates (108), resulting in eight IGFBP genes. It is thought that two of them were lost, resulting in the six types of IGFBPs seen in mammals and most other vertebrate classes (Figure 3). In many teleost fish, another round of tetraploidization occurred, resulting in a further doubling of IGFBP genes (16, 109). Some of these additional duplicates were subsequently retained in modern fish. Indeed, there is substantial variation in numbers of IGFBP genes between fish species (Table 1). The salmonid fish, whose common ancestor underwent a fourth round of whole genome duplication, exhibit the largest known repertoire of 22 IGFBP genes (22, 23). The preservation and evolutionary conservation of most of the IGFBP gene duplicates implies that these genes might have aquired unique evolutionarily adaptive roles, either by developing new functions opportunistically (neofunctionalization) or by retention of a subset of the parent gene’s original functions in each duplicate (subfunctionalization). This is in agreement with the idea that fine tuning of IGF signaling is strongly adaptive to the extent that perhaps even small changes in the regulation of IGF signaling would be sufficient to account for the conservation of additional IGFBP genes to provide these regulatory advantages.

The acquisition of IGF-independent actions of IGFBPs presents an intriguing question. One possible explanation is that they were present in the ancestral IGFBP gene. A comparative study suggested that the single amphioxus IGFBP has a functional nuclear localization sequence and transactivation activity but lacks the ability to bind modern IGFs (77). Both IGF-dependent and IGF-independent actions appear to have been present in the earliest vertebrates as indicated by the fact that an IGFBP from sea lamprey exhibited both IGF-dependent and -independent actions (110). Therefore, the IGF-binding function of IGFBPs may have been acquired later in evolution.

## Concluding Remarks and Prospects

**Figure 4:**
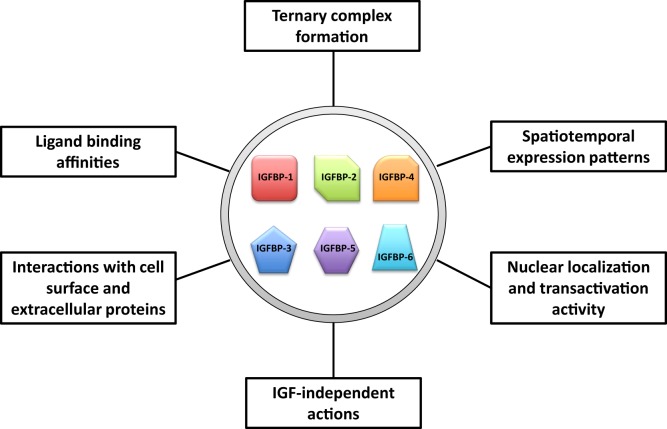
Major attributes of insulin-like growth factor-binding proteins (IGFBPs) that may help to give rise to the increased flexibility and versatility in their abilities to regulate insulin-like growth factor (IGF) actions.

We propose that IGFBPs provide a set of tools with which evolution has acted to increase the flexibility and versatility in the regulation of the IGF signaling system. An ancestral IGFBP gene has diversified into a number of IGFBP genes, which have both overlapping and unique expression patterns and functions. These IGFBPs can be viewed as different tools that all apply leverage but also provide individual context specific advantages. A number of attributes of IGFBPs may help to give rise to the increased flexibility and versatility in their abilities to regulate IGF actions. These include: (1) distinct spatiotemporal expression patterns of these IGFBP genes, (2) differences in their ligand-binding affinity and selectivity, (3) different roles in the circulation including formation of the ternary complex with ALS, (4) different abilities to interact with cell surface proteins, extracellular proteins, and other growth factors, (5) different subcellular localization, and (6) various IGF-independent activities (Figure 4). The existence of multiple IGFBPs can contribute to the fine-tuning of IGF signaling both globally and locally, and under various physiological and pathological conditions. The involvement of IGFBPs in mammary gland growth, liver regeneration, and atherogenesis, and the adaptive proliferation of calcium ionocytes in zebrafish are all examples of this sort of process. It is plausible that more IGFBPs will be found to participate in other roles of this type. A great deal of work has identified many roles for IGFBPs in cancer cells despite the fact that IGFBPs are not commonly mutated in human cancers (12). Given the involvement of IGFBPs in tissue remodeling and conditional proliferation of certain cell types, it is not surprising that their physiological actions would be coopted by cancer cells in order to facilitate the needs of tumor growth.

Much has been learned in recent decades about the cell type-specific actions of IGFBPs but many questions remain unanswered. One major question is, why do several of the IGFBPs have the ability to enter the cell nucleus? Although certain IGFBPs have a functional nuclear localization motif and a transactivation domain that are both evolutionarily conserved, the physiological functions of the nuclear IGFBPs remain unknown. Another area of inquiry for future research will be to identify additional stressful conditions that IGFBPs have evolved to respond to. It will also be of great interest to identify pathological processes that depend on the misregulation of IGFBP(s) to increase or decrease IGF signaling, or on inappropriate activation of their IGF-independent actions. We also have much more to learn regarding the evolutionary history of the IGFBPs in early vertebrates and the nature of its IGF-independent functions. This may shed light on the complex biology of modern IGFBPs.

CRISPR/Cas9-based genetic editing technology will allow the generation of mutant animals whose endogenous IGFBP genes are directly mutated to disrupt individual functionalities such as IGF-binding, nuclear translocation, or interaction with cell surface proteins, to allow assessing the roles of those capabilities individually or collectively under physiological conditions *in vivo*. The CRISPR-Cas9-based approaches will allow the physiological roles of redundant paralogs to be determined much more readily by enabling the generation of multiple knockouts at reasonable cost. Increasing our understanding of IGFBPs will yield insights into the array of biological processes to which IGF signaling is linked, including many that are crucial for human health and diseases.

## Author Contributions

CD conceived the article. JA and CD wrote the manuscript.

## Conflict of Interest Statement

The authors declare that the research was conducted in the absence of any commercial or financial relationships that could be construed as a potential conflict of interest.
